# Prospective associations of COVID-related stress with vaping nicotine and cannabis among high school students: Mediated by vaping susceptibility

**DOI:** 10.1371/journal.pone.0334159

**Published:** 2025-10-07

**Authors:** Ryan Lee, Junhan Cho, Dayoung Bae, Larisa Albers, Shirin Emma Herzig, Carla Michelle Ramirez, Alberto Carvajal, Daniel Soto, Jennifer B. Unger

**Affiliations:** Department of Population and Public Health Sciences, Keck School of Medicine, University of Southern California, Los Angeles, California, United States of America; National Institutes of Health, University of the Philippines Manila / De La Salle University, PHILIPPINES

## Abstract

Adolescent e-cigarette and cannabis vaping have become significant public health concerns, with rates increasing in recent years. However, there is limited research on the impacts of COVID-related stress on adolescent vaping. This study examined the longitudinal impacts of COVID-related stress on adolescent e-cigarette and cannabis vaping, including the mediating role of vaping susceptibility (which measures a lack of a firm commitment not to use a substance). We examined the prospective associations of COVID-related stress during remote learning (2020–2021) with e-cigarette and cannabis vaping use two years later (2022–2023) through the mediation of vaping susceptibility (2021–2022) among a cohort of students recruited as ninth graders from nine public high schools across Los Angeles County and surveyed annually (N = 1,316). Higher levels of COVID-related stress were prospectively associated with increased susceptibility to vaping e-cigarettes (B = 0.04, p = .02) and cannabis (B = 0.04, p = .02) one year later, which in turn increased the odds of e-cigarette (B = 0.98, p = .003) and cannabis (B = 1.62, p < .001) vaping two years later. This study highlights the critical need for effective, school-based prevention programs to reduce susceptibility to vaping, particularly during periods of heightened stress or future crises.

## Introduction

### Adolescent e-cigarette and cannabis use

Adolescent use of electronic nicotine delivery systems (ENDs), also known as e-cigarette use or vaping, is a significant public health concern. In 2020, 34.5% of United States (US) twelfth-grade students reported past month e-cigarette use [1]. Several studies have illustrated the negative impact of e-cigarette use on adolescent health [2–4]. The Centers for Disease Control and Prevention (CDC) has also outlined the role of nicotine on the developing brain, including dysregulation of brain regions responsible for impulse control, learning, and mental health [5]. Further, studies of adolescent cohorts frequently show that early exposure to nicotine may lead to substance use disorders later in life [6,7].

Cannabis is among the top three most prevalent substances used among adolescents [8]. In 2020, 21.1% of US 12th grade students were current cannabis users and 43.7% had reported lifetime use [1]. This is an area of concern as adolescents who use cannabis are less likely to graduate from high school or college, compared to those who do not use cannabis [9,10]. Adolescent cannabis use has also been associated with hindered neurodevelopment, increased addiction and comorbid substance use, suicidality, and new-onset psychosis [11,12]. The rise of adolescent cannabis vaping is also an area of concern with rates of current cannabis vaping almost doubling from 7.5% to 14% between 2018–2019 among US twelfth-grade students [1]. A recent meta-analysis found preference for cannabis products among adolescents may be shifting from dried herb to cannabis oil for vaping [13]. This is concerning because the rapid rise in e-cigarette vaping among adolescents may also be influencing cannabis vaping rates, as e-cigarette use has been found to be a significant risk factor for cannabis vaping initiation [14].

Given the rise in e-cigarette and cannabis vaping rates among adolescents, additional research on the risk and protective factors for vaping is warranted. For example, stressful life events are associated with tobacco and cannabis initiation and higher levels of tobacco and cannabis use among adolescents [15–17]. In addition to daily life stressors that adolescents face, stressors from extreme events, such as natural disasters, have also been associated with increased rates of substance use among adolescents [18,19]. The SARS-CoV-2 (COVID-19) pandemic caused extreme and daily life stressors for adolescents, including distance learning, social isolation, family economic losses, and potential family health deterioration or loss. The effects of the COVID-19 pandemic and its related stressors on adolescent substance use, however, are still not fully understood, especially their longitudinal impacts.

### Impact of the COVID-19 pandemic on adolescents

Adolescence is a life stage that is defined by significant social, physical, and cognitive transitions [20]. It is also a time in which adolescents begin to experiment with risky behaviors such as substance use. Life changes that occurred during the beginning phase of the COVID-19 pandemic may have added additional stress to the already complex lives of adolescents [21]. These COVID-related stressors changed the risk and protective factors for adolescent substance use during this time [22]. Previous studies have also found relationships between various COVID-related stressors and substance use; however, these studies were either cross-sectional or took place early in the pandemic and did not capture the lasting impact that COVID-related stress had on adolescents multiple years later [23–25]. Interestingly, some studies also found no significant relationships between COVID-related stress and substance use among adolescents [26,27]. In fact, a review article examining the impact of COVID-19 on adolescent substance use highlights the mixed findings among studies published early in the pandemic [28]. Given these mixed findings from studies that took place early in the COVID-19 pandemic, longitudinal studies on adolescent substance use during and coming out of the pandemic are warranted.

### Substance use susceptibility

Susceptibility indicates a lack of a firm commitment not to use a substance, suggesting one might use if presented with the opportunity. Understanding adolescent susceptibility to e-cigarette and cannabis use is important as susceptibility has been shown to significantly predict cigarette, e-cigarette, and cannabis initiation [29–33]. While there is little research on the impact of COVID-related stress on susceptibility among adolescents, it is likely that various factors could have impacted their openness to using substances and thus susceptibility may be an important mediator to investigate following instances of extreme stress and subsequent substance use among adolescents.

### The present study

Given the limited research on the longitudinal impacts of COVID-related stress on adolescent substance use, we examined the prospective associations of COVID-related stress with e-cigarette and cannabis vaping use through the mediation of vaping susceptibility among a cohort of high school students in Los Angeles County, California. Data from this cohort of student were collected at key timepoints throughout the COVID-19 pandemic, specifically the 2020–2021 school year (T1), when schools were closed, the 2021–2022 school year (T2), the first year after schools reopened, and the 2022–2023 school year (T3), two years after schools reopened. We hypothesized that 1) COVID-related stress (T1) would be directly associated with both higher e-cigarette and cannabis vaping behaviors two years later (T3), and 2) COVID-related stress (T1) would be associated with higher vaping susceptibility (T2), which would in turn be associated with higher e -cigarette and cannabis vaping behaviors (T3).

## Materials and methods

### Participants

Participants were high school students enrolled in a prospective cohort study called the Trends in Tobacco Use Survey (TITUS). Participants were originally recruited as 9^th^ grade students from nine public high schools in Los Angeles County, California in 2019–2020 (n = 1890). Participants completed annual surveys asking about e-cigarette and cannabis vaping behaviors. The study sample includes data from the three most recent survey waves, labeled T1 (2020–2021) T2 (2021–2022), and T3 (2022–2023). The first wave of survey data (2019–2020) was omitted because we were interested in measuring COVID-stress, which was added in the second wave of the survey. Among study enrollees, 1316 students provided valid data for COVID-related stress and substance use outcomes, constituting the analytic sample. See S1 Fig for information on accrual and inclusion in this study’s analytic sample (n = 1316). To assess potential bias from excluding participants with missing follow up survey data, we tested for differences in sociodemographic characteristics between students included in vs. excluded from the primary analytic sample (S1 Table).

### Procedure

This study was approved by the University of Southern California Institutional Review Board and informed written consent was obtained (IRB study number: HS-18–00706). School and participant recruitment for the TITUS Study are previously described by Vassey et al [34]. In short, parents received paper and electronic consent forms and study descriptions. Students with written parental consent were provided with a youth assent form prior to participation. Students who agreed provided written youth assent prior to completing annual surveys on their student Chromebooks via REDCap, an online survey platform, and received a $5 gift card for completing the survey. At T1, participants completed the survey remotely due to COVID-19 school closures in which research staff proctored the survey during synchronous online classes. Both T2 and T3 surveys were administered in-person in classrooms after students returned from distance learning. The recruitment period for this study started on August 1^st^, 2019, and ended on May 30^th^, 2021. All study procedures have conformed to the principles embodied in the Declaration of Helsinki.

### Measures

#### COVID-related stress.

The primary predictor, COVID-related stress, was assessed in T1. The COVID Stress scale assessed various life changes during COVID school-closures including family changes, responsibility changes, time commitment changes, socialization changes, etc [35,36]. See S2 Table for the COVID Stress scale items (S2 Table). Each item had response options of “yes” (1) or “no” (0). Each item was summed with 12 items reverse coded (Cronbach’s alpha = 0.75).

#### E-cigarette and cannabis vaping susceptibility.

Susceptibility to e-cigarette and cannabis vaping were the primary mediator variables and were assessed in T1 and T2. Susceptibility was assessed using adapted versions of Pierce et al.’s measure of smoking susceptibility [29]. Example items include, “Do you think you would use any of the following substances in the next year?” with response options from (1) definitely yes – (4) definitely not. Students who answered “definitely not” to all three questions were considered non-susceptible for that product; otherwise, students were considered susceptible. E-cigarette products included e-cigarette with nicotine, e-cigarette without nicotine, JUUL, and disposable e-cigarette device. Cannabis vaping products included electronic device to vape THC or hash oil.

#### Current E-cigarette & cannabis vaping use.

The primary outcome variables, current e-cigarette and cannabis vaping use, were assessed in T1 and T3 with the following survey question taken from the Youth Risk Behavior Surveillance System (YRBSS) Survey [37]: “In the past 30 days, how many total days have you used…?” [with response options to the same e-cigarette products listed above and cannabis vaping products listed above], (0 days, 1–2 days, 3–5 days, 6–9 days, 10–19 days, 20–29 days, all 30 days). E-cigarette use and vaping cannabis were coded as a binary variable (“yes” if one or more of these products was used for at least 1–2 days, and “no” if none of these products were used in the past 30 days).

#### Covariates.

Covariates at T1 were selected to parse the relative contribution of COVID-related stress to e-cigarette and vaping cannabis use at follow-up. Sociodemographic characteristics, including age, biological sex (male/female), race/ethnicity (Hispanic/Asian/White/African American/Other), self-reported family financial status (pretty well off/ above average/ poor/ varied), and parental educational attainment (college graduate/less than college graduate) were assessed. Additionally, baseline (T1) measures of e-cigarette and cannabis vaping susceptibility and e-cigarette, and cannabis vaping use were controlled for in the models.

### Statistical analysis

Preliminary analyses were conducted by calculating descriptive statistics for the study variables at T1, T2, and T3. Next, a regression-based path analysis estimated the main direct association of T1 COVID-related stress with T3 past 30-day e-cigarette use, after controlling for baseline e-cigarette use and other covariates (and excluding the mediator variable).

**Fig 1:**
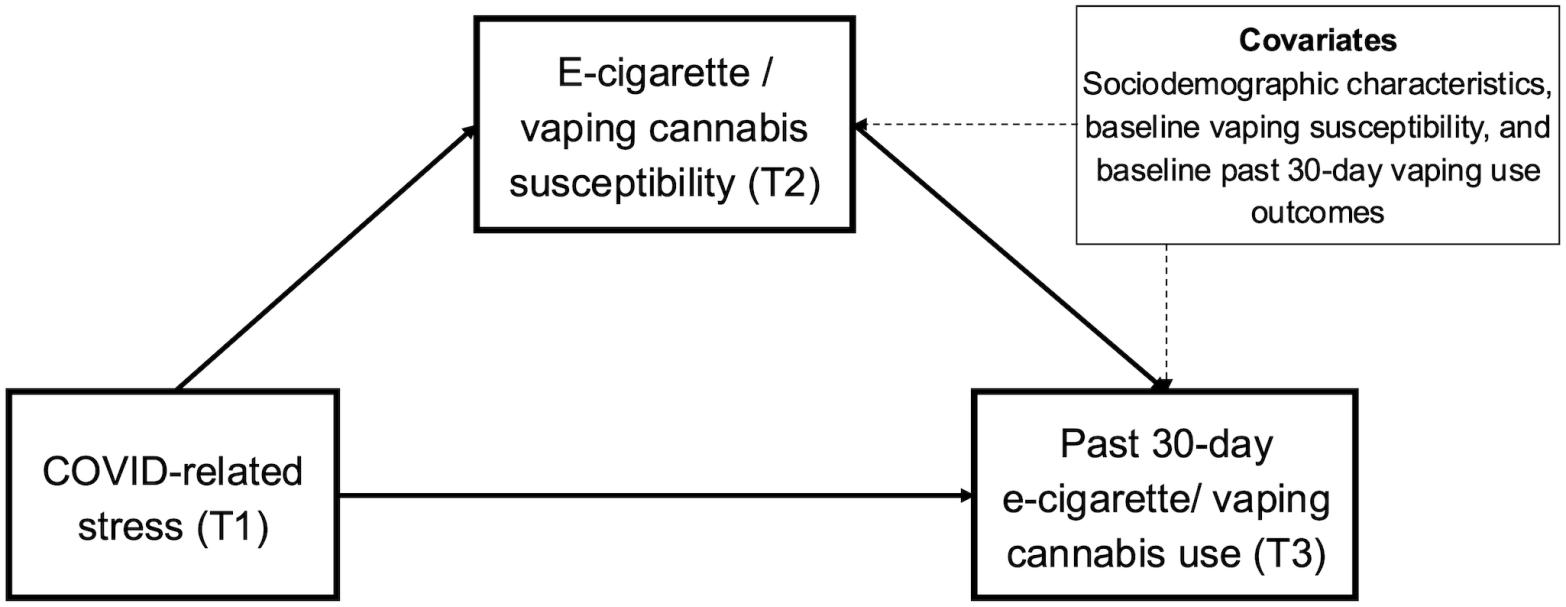
Visual schematic of path model. Note: baseline (T1) susceptibility, e-cigarette/cannabis vaping use, and other covariates (age, sex, race/ethnicity, family financial status, and parent education) were controlled in the mediational path model.

Next, we tested mediational process models by simultaneously including regression-based paths linking T1 COVID-related stress to T2 vaping susceptibility (Path a) and linking T2 vaping susceptibility to T3 past 30-day e-cigarette use outcome (Path b). The remaining direct effect path between T1 COVID-related stress and T3 e-cigarette use outcome (Path c’) was simultaneously modeled. Baseline (T1) vaping susceptibility, e-cigarette use, and other covariates (age, sex, race/ethnicity, family financial status, and parent education were controlled in the mediational path model. See Fig 1 for a visual schematic of the proposed path model highlighting the key study variables (See S4 and S5 Tables for all the pathways of our covariates in our primary models).

Finally, we tested indirect effects linking the T1 COVID-related stress to T3 e-cigarette via T2 vaping susceptibility using Monte Carlo integration methods. Effect sizes were calculated based on the mediation ratio—the proportion of indirect effect to the total effect. The same path analysis model was performed to test mediational pathways linking T1 COVID-related stress → T2 susceptibility for vaping cannabis → T3 past 30-day vaping cannabis. Binary logistic regression modeling of path analyses was used for past 30-day vaping outcome dichotomously coded. All models used standardized COVID-related stress scores (mean = 0, SD = 1) to facilitate interpretation and comparability of effect sizes.

Analyses were conducted in Mplus 8 using a complex modeling option, which adjusted parameter standard errors for interdependence in the data due to the nesting of students within schools (See S1 Appendix for Mplus code). Results of path coefficients are reported as standardized coefficients with 95% confidence intervals (95% CIs). Statistical significance was set at.05 (two-tailed). Missing data were addressed using a full information maximum likelihood estimation.

## Results

### Preliminary analyses

Descriptive statistics for demographics and study variables for the analytic sample are displayed in Table 1. The sample was 57.8% female and primarily Hispanic race/ethnicity (53.4% Hispanic). Among the analytic sample, 3.6% and 6.4% of participants reported e-cigarette use in the past 30 days at T1 and T3, respectively. Past 30-day cannabis vaping was 2.9% at T1 and 7.4% at T3. The average number of COVID-related stressors reported at T1 was 18.99 (*SD* = 4.93). The prevalence of vaping susceptibility at T2 was 38.4% for nicotine and 19.9% for cannabis, similar to the 36.7% for nicotine and 18.6% for cannabis reported at T1.

**Table 1:** Descriptive statistics.

Sociodemographic covariates, T1	N(%) or M(*SD*)^a^	Range
Female, N(%)	613 (57.8)	
Age (years), M(*SD*)	14.20 (0.44)	13-16
Race/ethnicity, N(%)		
Hispanic	563 (53.4)	
Asian	225 (21.3)	
White	84 (8.0)	
African American	40 (3.8)	
Other^b^	142 (13.4)	
Family financial status, N(%)		
Pretty well-off financially	323 (30.8)	
About average	571 (54.5)	
Poor	46 (4.4)	
It varied	108 (10.3)	
Parent education, N(%)^c^		
College graduate	384 (40.5)	
Less than college graduate	563 (59.5)	
**Covid-related stressor, T1, M(*SD*)**	18.99 (4.93)	5-32
**Substance use susceptibility**		
E-cigarette susceptibility, T1, N(%)^d^	445 (36.7)	
E-cigarette susceptibility, T2, N(%)^d^	446 (38.4)	
Vaping cannabis susceptibility, T1, N(%)^e^	220 (18.6)	
Vaping cannabis susceptibility, T2, N(%)^e^	221 (19.9)	
**Past 30-day substance use outcomes**		
E-cigarette, T1, N(%)^f^	48 (3.6)	
E-cigarette, T3, N(%)^f^	84 (6.4)	
Vaping cannabis, T1, N(%)^g^	38 (2.9)	
Vaping cannabis, T3, N(%)^g^	98 (7.4)	

^a^Available data Ns of the denominators for percentages reported for categorical variables ranged from 947 to 1316 (missing data range = 0.0%−28.0%). ^b^Other race/ethnicity includes American Indian, Alaska Native, Native Hawaiian, Pacific Islander, multiracial, and other races. ^c^Students who did not respond to the survey question or who marked “don’t know” are not included in the denominator. ^d^Respondents who were susceptible to any of the following: e-cigarette with nicotine, e-cigarette without nicotine, JUUL, and disposable e-cigarette device. ^e^Respondents who were susceptible to vaping cannabis. ^f^Binary outcome of past 30-day use of e-cigarette products (yes/no). ^g^Binary outcome of past 30-day use of vaping cannabis (yes/no). Note: Time 1 (T1) occurred in 2020–2021, Time 2 (T2) occurred in 2021–2022, and Time 3 (T3) occurred in 2022–2023.

### Mediational process of substance use susceptibility linking COVID-related stress to past 30-day substance use

**Fig 2:**
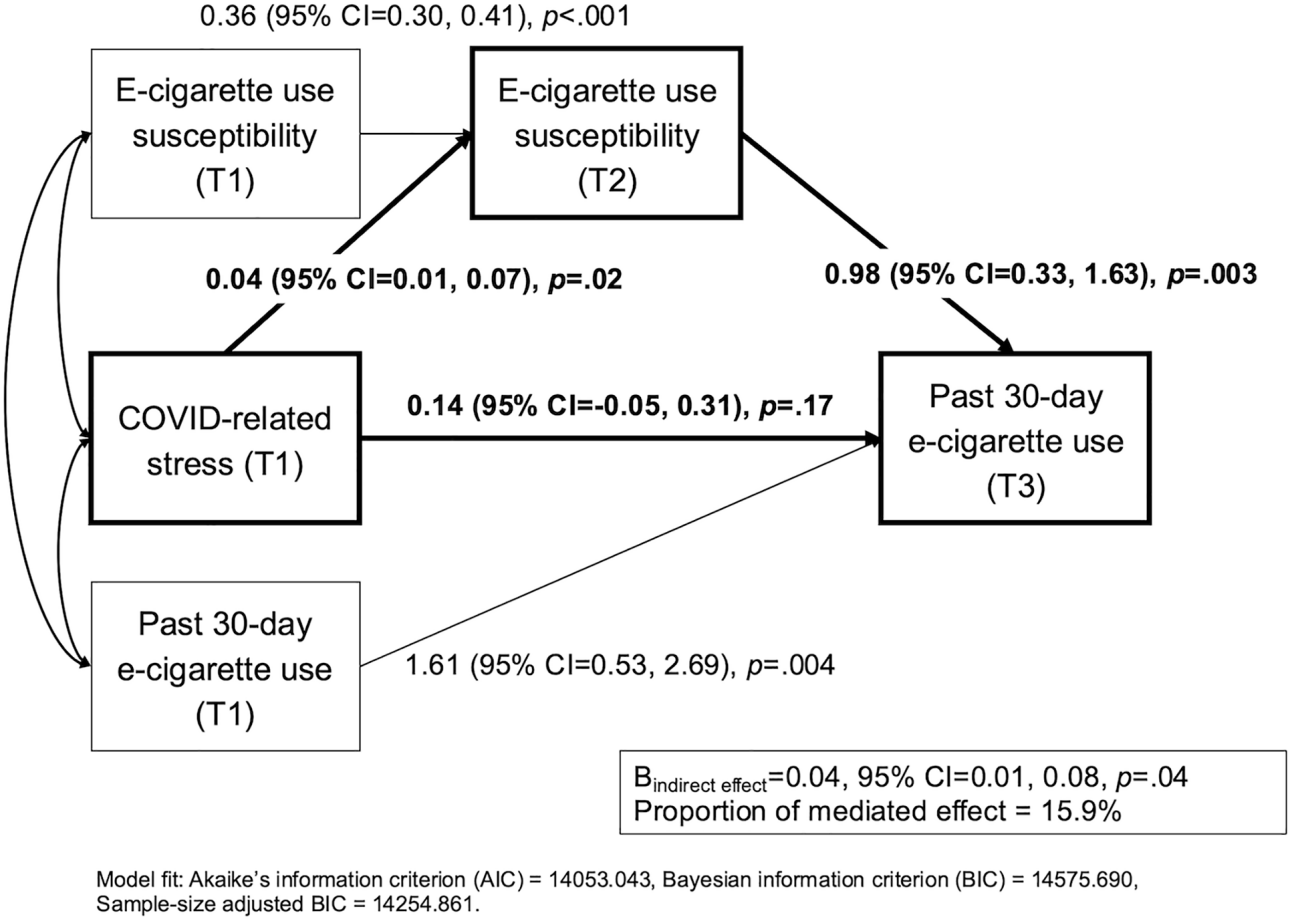
Indirect influence of COVID-related stress on past 30-day e-cigarette use via e-cigarette use susceptibility. *Note*: Standardized coefficients with 95% CIs and p-values are shown. Age, sex, race/ethnicity, family financial status, and parent education covariates were adjusted (see S4 Table).

A path model was first conducted to evaluate the total effect of COVID-related stress on past 30-day e-cigarette use. After controlling for T1 past 30-day e-cigarette use and other covariates, a one standard deviation increase in COVID-related stress at T1 was associated with a 24.4% increase in the odds of past 30-day e-cigarette use at T3 (2 years later; B [95%CI] = 0.21 [0.04, 0.40], *p* = .02; Odds Ratio[OR]=1.24 [1.04, 1.49]). As presented in Fig 2, we then performed a mediational path analysis to examine associations linking COVID stress (T1) to e-cigarette use (T3) via vaping susceptibility (T2). After adjusting for T1 vaping susceptibility and other covariates, a one standard deviation increase in T1 COVID-related stress was positively associated with increases in vaping susceptibility (B [95%CI] = 0.04 [0.01, 0.07], *p* = .02), which in turn significantly increased odds of e-cigarette use in past 30 days (B [95%CI] = 0.98 [0.33, 1.63], *p* = .003; OR=2.67 [1.39, 5.12]). The remaining direct effect of T1 COVID stress on T3 past 30-day e-cigarette use became non-significant (B [95%CI] = 0.14 [−0.05, 0.31], *p* = .17; OR=1.14 [0.95, 1.37]). The indirect effect of COVID stress T1 → Vaping susceptibility T2 → Past 30-day e-cigarette use T3 was significant (B_indirect effect_ [95%CI] = 0.04 [0.01, 0.08], *p* = .04), and the proportion of this mediated effect was 15.9%.

**Fig 3:**
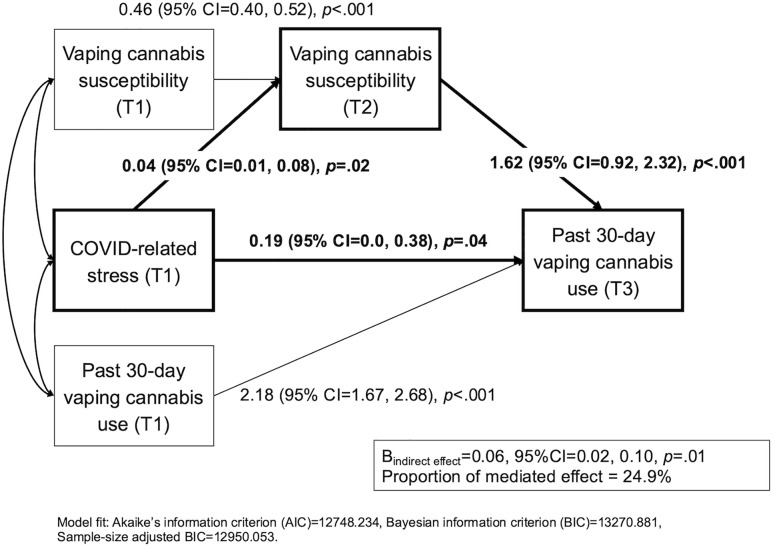
Indirect influence of COVID-related stress on past 30-day vaping cannabis use via vaping cannabis susceptibility. *Note*: Standardized coefficients with 95% CIs and p-values are shown. Age, sex, race/ethnicity, family financial status, and parent education covariates were adjusted (see S5 Table).

For the past 30-day vaping cannabis outcome, we also conducted a path model to examine the total main effect of COVID-related stress on past 30-day vaping cannabis. A one standard deviation increase in COVID-related stress at T1 was associated with higher odds of past 30-day vaping cannabis at T3 (2 years later; B [95%CI] = 0.26 [0.09, 0.43], *p* = .002; OR = 1.30 [1.10, 1.54]) after adjusting for T1 past 30-day vaping cannabis and other covariates. The mediational path analysis presented in Fig 3 indicates that a one standard deviation increase in T1 COVID-related stress significantly increased the risk for cannabis vaping susceptibility at T2 (B [95%CI] = 0.04 [0.01, 0.08], *p* = .02), which was positively associated with increased odds of past-30-day cannabis vaping at T3 (B [95%CI] = 1.62 [0.92, 2.32], **p* *< .001; OR = 5.04 [2.50, 9,08]). The remaining direct effect of T1 COVID-related stress on T3 past 30-day vaping cannabis was still significant (B [95%CI] = 0.19 [0.01, 0.38], *p* = .04; OR = 1.21 [1.01, 1.46]). The indirect effect of COVID stress T1 → Vaping susceptibility T2 → Past 30-day vaping cannabis T3 was significant (B_indirect effect_ [95%CI] = 0.06 [0.02, 0.10], *p* = .01), and proportion of this mediated effect was 24.9%. All path coefficients of mediational models are presented in S3 Table.

## Discussion

Emerging research on the impact of COVID-related stressors on adolescent substance use has yielded mixed results, and most of these studies took place early in the pandemic [23–28]. Our results offer new insights into the longitudinal impact of COVID-related stress on adolescent vaping behaviors. These results support our hypothesis and build upon previous literature, suggesting that COVID-related stress is positively and prospectively associated with adolescent substance use [15–19,23–25]. Although the COVID-19 pandemic has ended, research examining its impact on adolescents – who were in a critical stage of development during that time – remains essential for informing long-term interventions to reduce the risks of substance use in this vulnerable population.

This study is also among the first to examine vaping susceptibility as a mediator between COVID-related stress and future substance use among adolescents. While susceptibility has been linked to substance use initiation, few studies have explored this within the context of the COVID-19 pandemic [29–33]. One study found higher odds of e-cigarette susceptibility among on-campus 6th graders compared to remote learners during Spring 2021 [38], but none have assessed the mediating role of susceptibility in relation to COVID-related stress. Our findings suggest that interventions to reduce vaping susceptibility, particularly during times of extreme stress, are crucial for preventing future substance use.

It is also notable that the proportion of mediating effects ranged from 16% − 25%, suggesting the remaining observed effects between COVID-related stress and e-cigarette and cannabis vaping use two years later may be due to other mediating factors or a result of adolescents “skipping” susceptibility and proceeding directly to use. Given the high degree of change students experienced transitioning back to in-person schooling, it is possible there was a lagged effect during the first year back to school and there was a “jump” to more pre-pandemic levels of substance use in the second year back as high schoolers lives returned to “normal”. Additionally, the mediating effect of susceptibility may have been larger for cannabis vaping compared to e-cigarette use because e-cigarettes have become more “normalized” among adolescents and thus adolescents may not stay in a susceptible stage for very long. In contrast, cannabis vaping may be perceived as more harmful or taboo due to the status of cannabis as an illegal drug on a federal level and therefore, adolescents may stay susceptible to cannabis vaping for a longer period of time before becoming a cannabis vaping user. Additional research examining how harm perception impacts e-cigarette and cannabis vaping susceptibility and subsequent use is warranted.

Our findings also highlight the importance of substance use prevention programs, especially during times of stress. While there are numerous school-based substance use prevention programs, the need to continue this work is ever-lasting, especially in response to the rapidly changing landscape of adolescent substance use and rise of both nicotine and cannabis vaping [39–41]. While efforts have been made to add e-cigarette/vaping use materials to existing programs, only few have been evaluated [42]. It is also likely that students did not receive their existing substance use prevention programming while they were distance learning during COVID. This may have contributed to decreased knowledge, attitudes and refusal skills for these students. Thus, additional efforts are needed to ensure students receive effective and engaging substance use prevention programming during and following times of significant stressful life events. This also highlights the importance of e-learning, remote health programs, and digital health interventions, which can serve as tools to improve access to crucial information that can include stress regulation training, virtual peer support groups, and teach coping and refusal skills remotely, especially among students who do not attend in-person school or for times when schools are shut down.

### Limitations

Although this study highlights the lasting impact of COVID-related stress on the substance use of adolescents, there are several limitations that should be noted. All data were self-reported by participants, which is subject to recall biases. Due to the longitudinal nature of this survey-based study, some students were excluded from the analysis because of missing survey information between surveys. Analysis of differences in sociodemographic characteristics between students included in and excluded from the primary analytic sample found differences in biological sex and race/ethnicity between the groups. This should be considered carefully when interpreting the results of the study. Additionally, the nature of the COVID-19 pandemic and related school disruptions fluctuated over the course of this study (for example, despite schools reopening for in-person learning Fall 2021 (T2 of this study), the SARS-CoV-2 Omicron variant wave occurred November 2021), which likely introduced heterogeneity in exposure timing and intensity. Thus, real-world timing of COVID stress, susceptibility development, and vaping initiation may have overlapped more fluidly, potentially weakening causal inference. The study sample consisted of high school students from nine public high schools in Southern California, which may not be representative or generalizable to other populations including youth not attending school-based learning and youth in other states or regions with different demographic or sociopolitical makeups. The past 30-day e-cigarette and cannabis vaping rates in our sample of 12^th^ graders in T3 (6.4% and 7.4%, respectively) were lower than the national average among 12^th^ graders in the same year (2023) (16.9% and 13.7%, respectively) [1]. However, the e-cigarette rates were similar to the rates in California (7.6%) in the same year (2023) [1]. The authors could not find current rates of adolescent cannabis vaping in California. The most recent study found 9.8% of Los Angeles 12^th^ grade students vaped cannabis in the past 30-days in 2017 [43]. This highlights the variation in adolescent substance use across the United States and need for additional research and interventions in areas of high use. Notably, the proportion of mediated effects was relatively moderate. To assess the potential impact of unmeasured confounding, we calculated E-values in our primary mediational path models [44]. Unmeasured confounders would need moderate-to-large associations (E-value: 1.79–9.55; lower CI: 1.28–4.44) with both exposure and outcome to explain the observed associations. While these values suggest a degree of robustness, we acknowledge that moderate confounders—such as peer or parental substance use, mental health history, or contextual exposures—could still introduce bias. Therefore, the findings should be interpreted with consideration of their potential presence and plausibility.

In summary, the key limitations in this study include: (1) unmeasured confounders, (2) non-random attrition, (3) regional specificity, (4) self-report biases, (5) possible overlap in timing of stress and behavior, and (6) scale development without external validation.

### Conclusions

Life changes that occurred during the beginning phase of the COVID-19 pandemic may have added additional stress to the already complex lives of adolescents. Our results demonstrate higher levels of COVID-related stress during the early stage of the pandemic and when students were distance learning are associated increased risk of vaping susceptibility a year later, which in turn was associated with increased e-cigarette and cannabis vaping use a year later. Intervention efforts in high schools to mitigate vaping susceptibility, and subsequent vaping use, are warranted, especially in times of unprecedented stress like a future pandemic or natural disaster.

## Supporting information

S1 Fig Study accrual flow chart. (TIFF)

S1 File Plain Language Summary. (DOCX)

S2 File Codebook. (XLSX)

S1 Table Descriptive statistics of study covariates of students included (vs. excluded from) the primary analytic sample. (DOCX)

S2 Table Survey questionnaire for the COVID-Stress Scale. (DOCX)

S3 Table Mediational process of substance use susceptibility linking COVID-related stress to past 30-day substance use. (DOCX)

S4 Table Unstandardized parameter estimates for all paths from structural equation model of COVID-related stress, e-cigarette use susceptibility, and e-cigarette use. (DOCX)

S5 Table Unstandardized parameter estimates for all paths from structural equation model of COVID-related stress, vaping cannabis use susceptibility, and vaping cannabis use. (DOCX)

S6 Table Joint display showing COVID-stress quartiles, susceptibility prevalence at T2, and use prevalence at T3. (DOCX)

S1 Appendix Mplus Code. (DOCX)

S1 Dataset vapinguse. (XLS)
