# Supplementary figures and images for: Prospective associations of COVID-related stress with vaping nicotine and cannabis among high school students: Mediated by vaping susceptibility
Source: PLoS One. 2025 Oct 7;20(10):e0334159. doi: 10.1371/journal.pone.0334159 (PMC12503344; doi:10.1371/journal.pone.0334159)

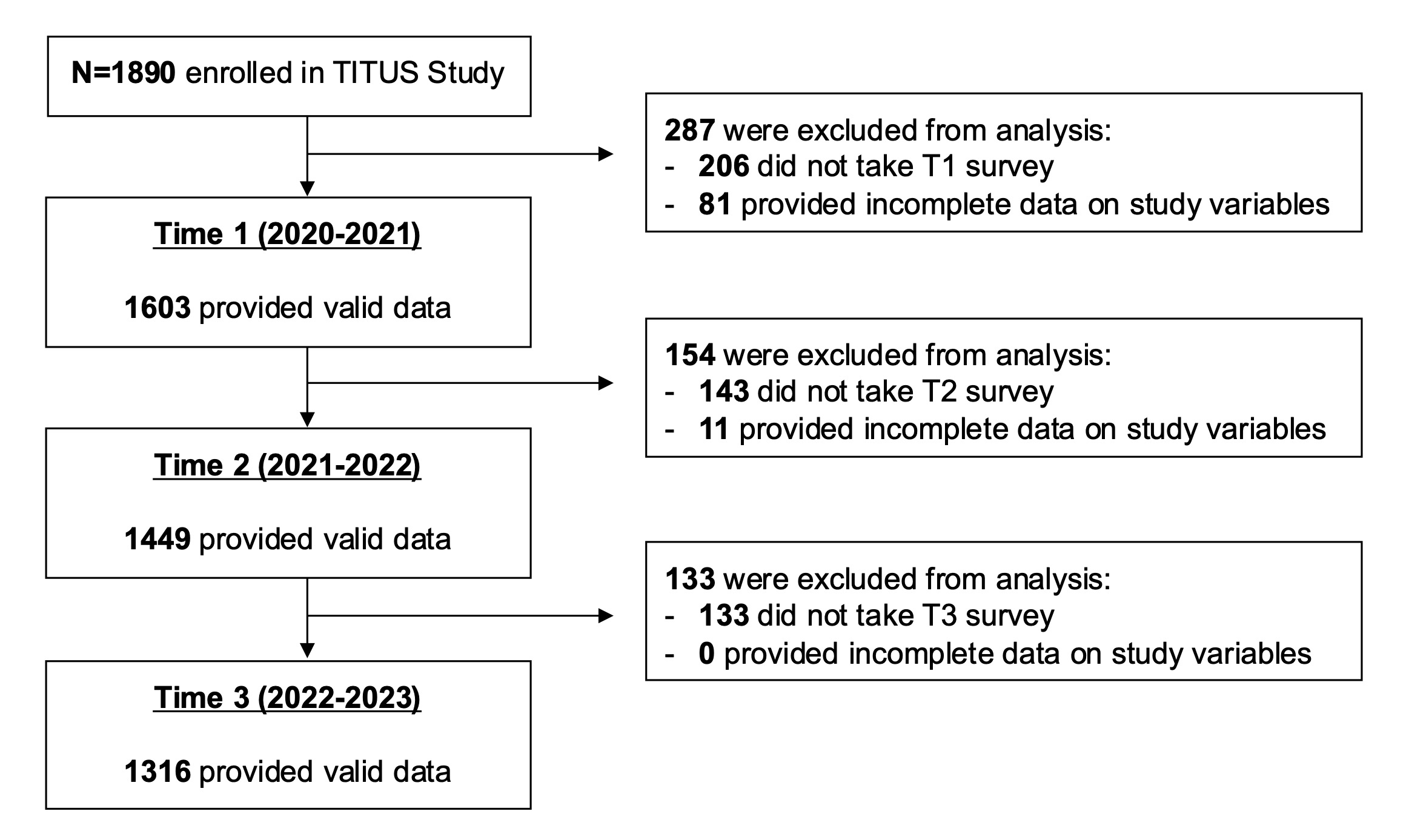

Supplement: S1 Fig — (TIFF) [file pone.0334159.s001.tiff]
