# Supplementary material for: Prospective associations of COVID-related stress with vaping nicotine and cannabis among high school students: Mediated by vaping susceptibility
Source: PLoS One. 2025 Oct 7;20(10):e0334159. doi: 10.1371/journal.pone.0334159 (PMC12503344; doi:10.1371/journal.pone.0334159)
Supplement: S1 File — (DOCX) [file pone.0334159.s002.docx]

**S1 File. Plain Language Summary**

**Study Title:**

Prospective associations of COVID-related stress with vaping nicotine and cannabis among high school students: Mediated by vaping susceptibility

**Background**

In recent years, rates of teen e-cigarette and cannabis vaping have increased. This has become a serious concern for public health. While many studies have looked at why teens vape, we still don’t know much about how stress from the COVID-19 pandemic may have influenced this behavior.

**What This Study Looked At**

We followed over 1,300 students from nine public high schools in Los Angeles County and surveyed them every year from 9^th^ grade through 12^th^ grade. The survey focused on:

- Student substance use and opinions of substance use (specifically e-cigarette and cannabis vaping)
- How much stress students felt during remote learning in 2020–2021 (at the height of the COVID-19 pandemic),

**Key Findings**

- **More stress = more risk**: Students who felt more COVID-related stress were more likely to become open to trying vaping—both e-cigarettes and cannabis.
- **Openness led to use**: This increased openness (or *susceptibility*) to vaping made it more likely that these students would actually start vaping later on.

**Why This Matters**

The study shows that stressful events like the COVID-19 pandemic can have long-lasting effects on teens' health choices. It highlights the importance of school-based prevention programs—especially ones that help teens manage stress and resist the temptation to start vaping.

**Takeaway**

Helping teens build resilience and make strong decisions about substance use is especially important during times of crisis. Schools and communities should be ready to support youth in times of crisis by ensuring access to important health programs whether in-person or remote.
