# Supplementary material for: Prospective associations of COVID-related stress with vaping nicotine and cannabis among high school students: Mediated by vaping susceptibility
Source: PLoS One. 2025 Oct 7;20(10):e0334159. doi: 10.1371/journal.pone.0334159 (PMC12503344; doi:10.1371/journal.pone.0334159)
Supplement: S1 Table — (DOCX) [file pone.0334159.s004.docx]

**S1 Table. Descriptive statistics of study variables of students** **included (vs. excluded from) in the primary analytic sample**

|  | **Analytic sample (N=1316)^a^** | **Excluded from the analysis across  follow-ups**  **(N=574)^b^** | **Group difference test^c^** |
| --- | --- | --- | --- |
|  | **M (SD) / N (%)** | **M (SD) / N (%)** | **P** |
| Female (vs. Male), N (%) | 613 (57.8) | 200 (46.7) | <.001 |
| Age, M (*SD*), year | 14.20 (0.44) | 14.22 (0.47) | n.s |
| Race/ethnicity, N (%) |  |  | <.001 |
| Hispanic | 563 (53.4) | 222 (52.5) |  |
| Asian | 225 (21.3) | 40 (9.5) |  |
| African American | 40 (3.8) | 13 (3.1) |  |
| Non-Hispanic White | 84 (8.0) | 79 (18.7) |  |
| Other^d^ | 142 (13.4) | 69 (16.2) |  |
| Above average family financial status (vs. poor and it varied), N(%) | 894 (85.3) | 148 (87.1) | n.s |
| Parents graduated college (vs. less education), N (%)^e^ | 384 (40.5) | 78 (48.8) | n.s |
| Covid-related stress | 18.99 (4.93) | 18.50 (7.78) | n.s |
| E-cigarette use, N(%) | 48 (3.6) | 6 (3.5) | n.s |
| Vaping cannabis use, N(%) | 38 (2.9) | 4 (2.3) | n.s |

Note. ^a^Available data (Ns=947-1316). ^b^Available data (Ns=428-ㅌㅌㅌ). ^c^Calculated using independent t-test for continuous variables and χ2 test for categorical variables. ^d^Other race/ethnicity includes American Indian, Alaska Native, Native Hawaiian, Pacific Islander, multiracial, and other races. ^e^Students who did not respond to the survey question or who marked “don’t know” are not included in the denominator.
