# Supplementary material for: Prospective associations of COVID-related stress with vaping nicotine and cannabis among high school students: Mediated by vaping susceptibility
Source: PLoS One. 2025 Oct 7;20(10):e0334159. doi: 10.1371/journal.pone.0334159 (PMC12503344; doi:10.1371/journal.pone.0334159)
Supplement: S2 Table — (DOCX) [file pone.0334159.s005.docx]

**S2 Table. Survey questionnaire for the COVID-Stress Scale**

Has COVID-19 changed your daily life in any of the following ways? (Response options: Yes=1, No=0)

| I couldn't do sports, school clubs, or other extracurricular activities |
| --- |
| I spent more time on screens and devices (e.g., looking at phone, playing video games, watching TV) |
| I had to stop going to places where I like to hang out (mall, park, restaurants, etc.) |
| *I got to do things I don't usually have time for (art, music, writing, cooking) |
| I couldn't see my friends |
| *I had better relationships with friends |
| I had celebrations (birthday parties, holiday parties) that were cancelled |
| I had to take care of siblings or other family members |
| I had more arguments or fights with my parents/guardians, siblings, or others who live with me |
| *I had a better relationship with family |
| My parent or guardian lost their job |
| My parents/guardians couldn't pay the bills |
| I was worried about the health of family members |
| I did not have enough freedom, privacy, or personal space |
| A family member used more alcohol, nicotine, or other substances |
| I had more responsibilities at home (chores, etc.) |
| *I had more time to relax |
| I did less physical activity or exercise |
| I overate or ate more unhealthy foods (e.g., junk food) |
| *I got more control to make my own schedule |
| I saw too much bad news in the media |
| *I spent more time doing enjoyable activities (e.g., reading books, puzzles) |
| I was bored at home |
| *I had more time to exercise or go outside |
| *I got more sleep |
| *I spent more time with my pet(s) |
| I felt lonely or isolated |
| I felt anxious, stressed, or depressed |
| It was hard to adjust to doing schoolwork at home |
| I missed seeing my teachers |
| I fell behind on schoolwork |
| *I had less schoolwork |
| *I had less stress/pressure from school and activities |
| *I experienced less bullying or arguments with other kids |
| I had school events (Prom, graduation, parties, field trips) that were cancelled |

*Items that were reverse coded.
