# Supplementary material for: Prospective associations of COVID-related stress with vaping nicotine and cannabis among high school students: Mediated by vaping susceptibility
Source: PLoS One. 2025 Oct 7;20(10):e0334159. doi: 10.1371/journal.pone.0334159 (PMC12503344; doi:10.1371/journal.pone.0334159)
Supplement: S3 Table — (DOCX) [file pone.0334159.s006.docx]

**S3 Table.** **Mediational process of substance use susceptibility linking COVID-related stress to past 30-day substance use**

| **Total effect** | | **Mediational process** | | | | | | **Indirect effect** | |
| --- | --- | --- | --- | --- | --- | --- | --- | --- | --- |
| COVID stress →  E-cigarette use  (Path c) | | COVID stress →  E-cigarette susceptibility (Path a) | | E-cigarette susceptibility →  E-cigarette use  (Path b) | | COVID stress →  E-cigarette use  (Path c’) | | COVID stress →  E-cigarette susceptibility →  E-cigarette use | |
| B (95% CI) | P | B (95% CI) | P | B (95% CI) | P | B (95% CI) | P | B (95% CI) | P |
| 0.21 (0.04, 0.40) | .02 | 0.04 (0.01, 0.07) | .02 | 0.98 (0.33, 1.63) | .003 | 0.14 (-0.05, 0.31) | .17 | 0.04 (0.01, 0.08) | .04 |
| COVID stress →  Vaping cannabis use  (Path c) | | COVID stress →  Vaping cannabis susceptibility (Path a) | | Vaping cannabis susceptibility → Vaping cannabis use  (Path b) | | COVID stress →  Vaping cannabis use  (Path c’) | | COVID stress →  Vaping cannabis susceptibility → Vaping cannabis use | |
| B (95% CI) | P | B (95% CI) | P | B (95% CI) | P | B (95% CI) | P | B (95% CI) | P |
| 0.26 (0.09, 0.43) | .002 | 0.04 (0.01, 0.08) | .02 | 1.62(0.92, 2.32) | <.001 | 0.19 (0.01, 0.38) | .04 | 0.06 (0.02, 0.10) | .01 |

Note. B=Unstandardized path coefficient. 95% CI=95% confidence interval
