# Supplementary material for: Prospective associations of COVID-related stress with vaping nicotine and cannabis among high school students: Mediated by vaping susceptibility
Source: PLoS One. 2025 Oct 7;20(10):e0334159. doi: 10.1371/journal.pone.0334159 (PMC12503344; doi:10.1371/journal.pone.0334159)
Supplement: S4 Table — (DOCX) [file pone.0334159.s007.docx]

**S4 Table. Unstandardized parameter estimates for all paths from structural equation model of COVID-related stress, e-cigarette use susceptibility, and e-cigarette use**

| Estimated parameters | Estimate | 95% CI | *P* |
| --- | --- | --- | --- |
| *Directional paths between covariates and main study variables* | | | |
| E-cigarette use (T1) → E-cigarette use (T3) | 1.61 | 0.53, 2.69 | .004 |
| Sex (T1) → E-cigarette use (T3) | -1.08 | -1.56, -0.60 | <.001 |
| Age (T1) → E-cigarette use (T3) | -0.25 | -0.67, 0.17 | .24 |
| Family financial status → E-cigarette use (T3) | -0.11 | -0.37, 0.14 | .39 |
| Parental education → E-cigarette use (T3) | -0.16 | -1.06, 0.74 | .73 |
| Race: Asian (T1) → E-cigarette use (T3) | -1.22 | -1.78, -0.67 | <.001 |
| Race: White (T1) → E-cigarette use (T3) | -0.32 | -0.86, 0.23 | .26 |
| Race: African American (T1) → E-cigarette use (T3) | -0.22 | -2.04, 1.81 | .78 |
| Race: Other (T1) → E-cigarette use (T3) | -0.24 | -1.03, 0.54 | .54 |
| Susceptibility (T1) → Susceptibility (T2) | 0.36 | 0.30, 0.41 | .01 |
| Sex (T1) → Susceptibility (T2) | -0.07 | -0.12, -0.02 | .01 |
| Age (T1) → Susceptibility (T2) | -0.04 | -0.15, 0.06 | .43 |
| Family financial status → Susceptibility (T2) | -0.01 | -0.04, 0.01 | .30 |
| Parental education → Susceptibility (T2) | 0.05 | -0.02, 0.12 | .15 |
| Race: Asian (T1) → Susceptibility (T2) | -0.10 | -0.22, 0.02 | .11 |
| Race: White (T1) → Susceptibility (T2) | 0.01 | -0.09, 0.09 | .96 |
| Race: African American (T1) → Susceptibility (T2) | -0.09 | -0.16, -0.03 | .01 |
| Race: Other (T1) → Susceptibility (T2) | -0.06 | -0.15, 0.03 | .18 |
| *Non-directional paths among covariates* |  |  |  |
| E-cigarette use (T1) ↔ Susceptibility (T1) | 0.02 | 0.01, 0.03 | <.001 |
| E-cigarette use (T1) ↔ COVID-related stress (T1) | 0.01 | -0.01, 0.02 | .47 |
| E-cigarette use (T1) ↔ Family financial status (T1) | 0.01 | -0.01, 0.02 | .65 |
| E-cigarette use (T1) ↔ Parental education (T1) | -0.01 | -0.01, 0.01 | .11 |
| E-cigarette use (T1) ↔ Race: Asian (T1) | -0.01 | -0.01, 0.01 | .15 |
| E-cigarette use (T1) ↔ Race: White (T1) | 0.01 | -0.01, 0.01 | .17 |
| E-cigarette use (T1) ↔ Race: Other (T1) | -0.01 | -0.01, 0.01 | .47 |
| Susceptibility (T1) ↔ COVID-related stress (T1) | 0.08 | 0.06, 0.10 | <.001 |
| Susceptibility (T1) ↔ Sex (T1) | -0.02 | -0.03, -0.01 | .04 |
| Susceptibility (T1) ↔ Age (T1) | 0.02 | -0.01, 0.03 | .10 |
| Susceptibility (T1) ↔ Family financial status (T1) | 0.01 | -0.02, 0.03 | .69 |
| Susceptibility (T1) ↔ Parental education (T1) | -0.01 | -0.02, 0.01 | .22 |
| Susceptibility (T1) ↔ Race: Asian (T1) | -0.01 | -0.02, 0.01 | .72 |
| Susceptibility (T1) ↔ Race: African American (T1) | -0.01 | -0.01, 0.01 | .10 |
| Susceptibility (T1) ↔ Race: Other (T1) | 0.01 | -0.01, 0.02 | .27 |
| COVID-related stress (T1) ↔ Family financial status (T1) | 0.12 | 0.07, 0.18 | <.001 |
| COVID-related stress (T1) ↔ Parental education (T1) | -0.05 | -0.10, 0.01 | .09 |
| COVID-related stress (T1) ↔ Race: Asian (T1) | -0.03 | -0.04, -0.01 | .01 |
| COVID-related stress (T1) ↔ Race: White (T1) | -0.02 | -0.06, 0.01 | .16 |
| COVID-related stress (T1) ↔ Race: African American (T1) | -0.01 | -0.02, 0.01 | .09 |
| COVID-related stress (T1) ↔ Race: Other (T1) | 0.02 | 0.01, 0.03 | .01 |
| Sex (T1) ↔ Age (T1) | 0.02 | 0.01, 0.03 | .01 |
| Sex (T1) ↔ Family financial status (T1) | 0.01 | -0.02, 0.04 | .36 |
| Sex (T1) ↔ Parental education (T1) | 0.01 | -0.01, 0.02 | .44 |
| Sex (T1) ↔ Race: Asian (T1) | 0.01 | -0.01, 0.02 | .60 |
| Sex (T1) ↔ Race: White (T1) | 0.01 | -0.01, 0.01 | .30 |
| Sex (T1) ↔ Race: African American (T1) | -0.01 | -0.01, 0.01 | .09 |
| Sex (T1) ↔ Race: Other (T1) | -0.01 | -0.01, 0.01 | .67 |
| Age (T1) ↔ Parental education (T1) | 0.01 | -0.02, 0.03 | .69 |
| Family financial status (T1) ↔ Parental education (T1) | -0.08 | -0.09, -0.06 | .01 |
| Family financial status (T1) ↔ Race: Asian (T1) | -0.04 | -0.06, -0.02 | .01 |
| Family financial status (T1) ↔ Race: African American (T1) | -0.01 | -0.02, -0.01 | .02 |
| Family financial status (T1) ↔ Race: Other (T1) | -0.01 | -0.03, 0.01 | .08 |

*Note.* Unstandardized path coefficients are shown for estimated parameters. The directional paths of main study variables are not included but are presented in Table S3. Non-directional paths among covariates that were non-significant and do not improve the model fit were excluded for model parsimony.
