# Supplementary material for: Prospective associations of COVID-related stress with vaping nicotine and cannabis among high school students: Mediated by vaping susceptibility
Source: PLoS One. 2025 Oct 7;20(10):e0334159. doi: 10.1371/journal.pone.0334159 (PMC12503344; doi:10.1371/journal.pone.0334159)
Supplement: S5 Table — (DOCX) [file pone.0334159.s008.docx]

**S5 Table. Unstandardized parameter estimates for all paths from structural equation model of COVID-related stress, vaping cannabis use susceptibility, and vaping cannabis use**

| Estimated parameters | Estimate | 95% CI | *P* |
| --- | --- | --- | --- |
| *Directional paths between covariates and main study variables* |  | | |
| Vaping cannabis use (T1) → Vaping cannabis use (T3) | 2.18 | 0.92, 2.32 | <.001 |
| Sex (T1) → Vaping cannabis use (T3) | 0.02 | -0.55, 0.60 | .94 |
| Age (T1) → Vaping cannabis use (T3) | -0.22 | -0.57, 0.14 | .23 |
| Family financial status → Vaping cannabis use (T3) | -0.13 | -0.22, -0.04 | .01 |
| Parental education → Vaping cannabis use (T3) | -0.42 | -0.84, -0.01 | .04 |
| Race: Asian (T1) → Vaping cannabis use (T3) | -0.80 | -1.78, 0.19 | .11 |
| Race: White (T1) → Vaping cannabis use (T3) | -0.05 | -0.70, 0.61 | .87 |
| Race: African American (T1) → Vaping cannabis use (T3) | -0.06 | -1.31, 1.19 | .92 |
| Race: Other (T1) → Vaping cannabis use (T3) | -0.27 | -0.73, 0.22 | .27 |
| Susceptibility (T1) → Susceptibility (T2) | 0.46 | 0.40, 0.52 | <.001 |
| Sex (T1) → Susceptibility (T2) | -0.02 | -0.08, 0.04 | .46 |
| Age (T1) → Susceptibility (T2) | -0.02 | -0.06, 0.02 | .36 |
| Family financial status → Susceptibility (T2) | -0.01 | -0.27, 0.13 | .56 |
| Parental education → Susceptibility (T2) | 0.03 | -0.01, 0.07 | .15 |
| Race: Asian (T1) → Susceptibility (T2) | -0.10 | -0.18, -0.01 | .02 |
| Race: White (T1) → Susceptibility (T2) | 0.03 | -0.05, 0.11 | .50 |
| Race: African American (T1) → Susceptibility (T2) | -0.08 | -0.20, 0.03 | .16 |
| Race: Other (T1) → Susceptibility (T2) | -0.03 | -0.10, 0.04 | .38 |
| *Non-directional paths among covariates* |  |  |  |
| Vaping cannabis use (T1) ↔ Susceptibility (T1) | 0.03 | 0.02, 0.05 | <.001 |
| Vaping cannabis use (T1) ↔ COVID-related stress (T1) | 0.01 | -0.01, 0.02 | .08 |
| Vaping cannabis use (T1) ↔ Parental education (T1) | -0.01 | -0.01, -0.01 | .01 |
| Vaping cannabis use (T1) ↔ Race: Asian (T1) | -0.01 | -0.01, -0.01 | .02 |
| Vaping cannabis use (T1) ↔ Race: Other (T1) | -0.01 | -0.01, 0.01 | .69 |
| Susceptibility (T1) ↔ COVID-related stress (T1) | 0.05 | 0.01, 0.08 | .01 |
| Susceptibility (T1) ↔ Sex (T1) | 0.01 | -0.02, 0.01 | .19 |
| Susceptibility (T1) ↔ Age (T1) | -0.01 | -0.01, 0.01 | .49 |
| Susceptibility (T1) ↔ Parental education (T1) | -0.02 | -0.03, -0.01 | .01 |
| Susceptibility (T1) ↔ Race: White (T1) | -0.01 | -0.01, 0.01 | .46 |
| Susceptibility (T1) ↔ Race: African American (T1) | -0.01 | -0.01, -0.01 | .01 |
| Susceptibility (T1) ↔ Race: Other (T1) | 0.01 | -0.01, 0.01 | .32 |
| COVID-related stress (T1) ↔ Age (T1) | -0.03 | -0.05, -0.01 | .03 |
| COVID-related stress (T1) ↔ Family financial status (T1) | 0.13 | 0.07, 0.19 | .01 |
| COVID-related stress (T1) ↔ Parental education (T1) | -0.04 | -0.10, 0.01 | .10 |
| COVID-related stress (T1) ↔ Race: Asian (T1) | -0.02 | -0.04, -0.01 | .01 |
| COVID-related stress (T1) ↔ Race: White (T1) | -0.02 | -0.06, 0.01 | .16 |
| COVID-related stress (T1) ↔ Race: African American (T1) | -0.01 | -0.02, 0.01 | .09 |
| COVID-related stress (T1) ↔ Race: Other (T1) | 0.02 | 0.01, 0.03 | .02 |
| Sex (T1) ↔ Age (T1) | 0.02 | 0.01, 0.03 | .01 |
| Sex (T1) ↔ Family financial status (T1) | -0.01 | -0.02, 0.04 | .35 |
| Sex (T1) ↔ Parental education (T1) | 0.01 | -0.01, 0.02 | .42 |
| Sex (T1) ↔ Race: Asian (T1) | -0.01 | -0.02, 0.02 | .66 |
| Sex (T1) ↔ Race: White (T1) | 0.01 | -0.01, 0.01 | .31 |
| Sex (T1) ↔ Race: African American (T1) | -0.01 | -0.01, 0.01 | .08 |
| Sex (T1) ↔ Race: Other (T1) | -0.01 | -0.01, 0.01 | .52 |
| Age (T1) ↔ Parental education (T1) | 0.01 | -0.02, 0.03 | .64 |
| Family financial status (T1) ↔ Parental education (T1) | -0.08 | -0.09, -0.06 | .01 |
| Family financial status (T1) ↔ Race: Asian (T1) | -0.04 | -0.06, -0.02 | <.001 |
| Family financial status (T1) ↔ Race: White (T1) | -0.01 | -0.02, 0.01 | .66 |
| Family financial status (T1) ↔ Race: African American (T1) | -0.01 | -0.02, -0.01 | .01 |
| Family financial status (T1) ↔ Race: Other (T1) | -0.01 | -0.03, 0.01 | .08 |

*Note.* Unstandardized path coefficients are shown for estimated parameters. The directional paths of main study variables are not included but are presented in Table S3. Non-directional paths among covariates that were non-significant and do not improve the model fit were excluded for model parsimony.
