# Supplementary material for: Prospective associations of COVID-related stress with vaping nicotine and cannabis among high school students: Mediated by vaping susceptibility
Source: PLoS One. 2025 Oct 7;20(10):e0334159. doi: 10.1371/journal.pone.0334159 (PMC12503344; doi:10.1371/journal.pone.0334159)
Supplement: S6 Table — (DOCX) [file pone.0334159.s009.docx]

**S6 Table.** Joint display showing COVID-stress quartiles, susceptibility prevalence at T2, and use prevalence at T3.

| COVID-Stress  Quartile | % E-cigarette Susceptible at T2 | % E-cigarette use  at T3 | % Vaping cannabis susceptibility at T2 | % Vaping cannabis use at T3 |
| --- | --- | --- | --- | --- |
| sQ1 (lowest) | 26.2% | 4.9% | 12.2% | 3.9% |
| Q2 | 41.3% | 5.7% | 22.9% | 5.7% |
| Q3 | 43.5% | 7.7% | 20.3% | 9.5% |
| Q4 (highest) | 46.8% | 7.7% | 25.6% | 10.4% |
