# Supplementary material for: Prospective associations of COVID-related stress with vaping nicotine and cannabis among high school students: Mediated by vaping susceptibility
Source: PLoS One. 2025 Oct 7;20(10):e0334159. doi: 10.1371/journal.pone.0334159 (PMC12503344; doi:10.1371/journal.pone.0334159)
Supplement: S1 Appendix — (DOCX) [file pone.0334159.s010.docx]

**S1 Appendix: Mplus Code**

Title: !Explanation marks are used for comments.

Data: File is c:\data\vapinguse.csv;

Variable: names = id sch ecig1 ecig3 suscept1 suscept2 covidstress age sex famfin paredu asian white black other;

Missing = . ;

Usevariables = sch !School clusters

ecig1 !E-cigarette use at T1

ecig3 !E-cigarette use at T3

suscept1 !E-cigarette use susceptibility at T1

suscept2 !E-cigarette use susceptibility at T2

covidstress !COVID-related stress at T1

age !Age

sex !Sex

famfin !Family financial status

paredu !Parental education

asian !Race/ethnicity

white !Race/ethnicity

black !Race/ethnicity

other; !Race/ethnicity

Cluster = sch;

Categorical = ecig3;

Analysis: type=complex; estimator=mlr;

Model:

ecig3 on suscept2 covidstress ecig1 age sex famfin paredu asian white black other;

suscept2 on covidstress suscept1 age sex famfin paredu asian white black other;

ecig1 with suscept1 covidstress famfin paredu asian white other;

suscept1 with covidstress sex age famfin paredu asian black other;

covidstress with famfin paredu asian white black other;

sex with age famfin paredu asian white black other;

age paredu;

famfin with paredu asian white black other;

Output: sampstat cinterval;
